# Pleiotropic effects between cardiovascular disease risk factors and measures of cognitive and physical function in long-lived adults

**DOI:** 10.1038/s41598-021-97298-0

**Published:** 2021-09-09

**Authors:** Julia J. Yudkovicz, Ryan L. Minster, Emma Barinas-Mitchell, Kaare Christensen, Mary Feitosa, Megan S. Barker, Anne B. Newman, Allison L. Kuipers

**Affiliations:** 1 Department of Epidemiology, University of Pittsburgh, Pittsburgh, PA USA; 2 Department of Human Genetics, University of Pittsburgh, Pittsburgh, PA USA; 3 Department of Epidemiology, Biostatistics and Biodemography, Danish Aging Research Center, University of Southern Denmark, Odense C, Denmark; 4 Division of Statistical Genomics, Department of Genetics, Washington University School of Medicine, St. Louis, MO USA; 5 Department of Neurology, Columbia University, New York, NY USA

**Keywords:** Epidemiology, Genetics research

## Abstract

Cardiovacular disease (CVD) is the leading cause of death among older adults and is often accompanied by functional decline. It is unclear what is driving this co-occurrence, but it may be behavioral, environmental and/or genetic. We used a family-based study to estimate the phenotypic and shared genetic correlation between CVD risk factors and physical and cognitive functional measures. Participants (n = 1,881) were from the Long Life Family Study, which enrolled families based on their exceptional longevity (sample mean age = 69.4 years, 44% female). Cardiovascular disease risk factors included carotid vessel measures [intima-media thickness and inter-adventitial diameter], obesity [body mass index (BMI) and waist circumference], and hypertension [systolic and diastolic blood pressures]. Function was measured in the physical [gait speed, grip strength, chair stand] and cognitive [digital symbol substitution test, retained and working memory, semantic fluency, and trail making tests] domains. We used SOLAR to estimate the genetic, environmental, and phenotypic correlation between each pair adjusting for age, age^2^, sex, field center, smoking, height, and weight. There were significant phenotypic correlations (range |0.05–0.22|) between CVD risk factors and physical and cognitive function (all *P* < 0.05). Most significant genetic correlations (range |0.21–0.62|) were between CVD risk factorsand cognitive function, although BMI and waist circumference had significant genetic correlation with gait speed and chair stand time (range |0.29–0.53|; all *P* < 0.05). These results suggest that CVD risk factors may share a common genetic-and thus, biologic-basis with both cognitive and physical function. This is particularly informative for research into the genetic determinants of chronic disease.

## Introduction

Although cardiovascular disease (CVD) burden has recently been somewhat reduced through improved prevention and treatment, it remains highly prevalent and the leading cause of death within the United States among men and women who are 65 years or older^1–4^. Due to an aging population, the prevalence of dementia, frailty, and CVD will likely continue to increase. It is therefore important to investigate whether a biological link exists between these aging-related conditions.

Prior research has shown that a presence of CVD is associated with the level of cognitive and physical functioning^5–9^. For instance, various cross-sectional studies have indicated that carotid intima-media thickness (IMT) is negatively associated with working memory, psychomotor speed, delayed recall and executive functioning^5,6^. There is also evidence that having a cardiovascular condition may put an individual at risk for future functional decline. For example in longitudinal studies, both a larger IMT and mid-life hypertension are associated with declines in executive functioning, retained memory, and category fluency, as well as a higher incidence of dementia, as indicated by several longitudinal studies^10–16^.

Most of the studied confounders or mediators of the relationship between CVD and function are biologic and/or behavioral, such as age, sex, excess weight, smoking, depression, education level and inactivity. However, each of these conditions is heritable in families, with specific estimates ranging from 0.21 for IMT^17^ to 0.68 for inter-adventitial diameter (IAD)^18^, and estimates of heritability of BMI are 0.4–0.9^19–23^. This suggests that there could be genetic correlations that exist between these aging-related disorders, which are often not considered in studies of co- or multi-morbidity in older adults. Investigating the shared etiology between cognitive and physical function and CVD may help to elucidate the pathophysiology of these conditions or provide potential targets for preventative strategies for co-morbidities in older adults.

Therefore, the purpose of this study was to determine the extent to which CVD risk factors, including measures of subclinical carotid vessel disease, obesity, and hypertension, are not only phenotypically correlated, but also genetically correlated with cognitive and physical function in adults from families with exceptional longevity from the Long Life Family Study (LLFS). We hypothesized that CVD risk factors will demonstrate an inverse genetic correlation with measures of cognitive and physical function, adjusting for demographic and environmental covariates.

## Methods

### Long life family study (LLFS) cohort

The LLFS cohort is comprised of multigenerational families selected for exceptional longevity. The study recruited participants from three sites across the United States (Pittsburgh, PA; Boston, MA; New York, NY) and Denmark, beginning in 2005. Families were recruited for this study based on first identifying long-lived individuals (probands) and their similarly long-lived siblings from public records (where “long-lived” was defined as aged 80 + years in the US and 90 + years in Denmark). Families were then assessed as to whether or not they demonstrated exceptional survival based on the Family Longevity Selection Score (FLoSS), which is a summary measure of the survival experience for probands and their siblings relative to what would be expected based on birth cohort-specific life tables and the availability of living subjects for the study^24^. We also enrolled any interested siblings of the probands, as well as, all interested spouses and offspring.

**Figure 1:**
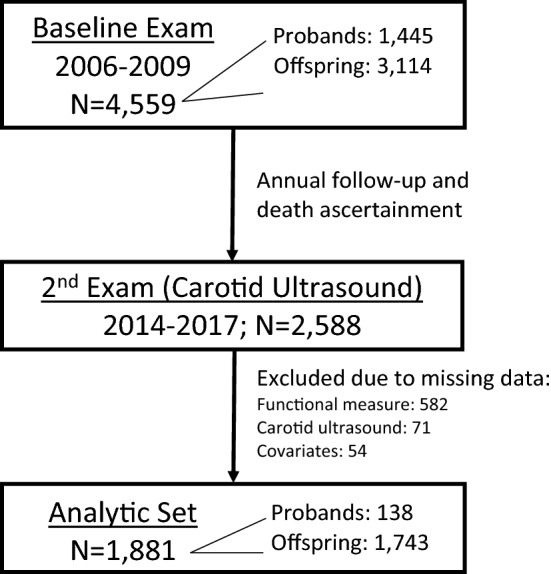
Flow-chart of Analytic Dataset from the Long Life Family Study. Depiction of the Long Life Family Study design, sample sizes, and exclusion criteria for the current study.

All LLFS data were collected via in-home examination. Some measures of interest, such as the carotid ultrasound, were only collected at the second visit conducted between 2014–2017 (N = 2588; Fig. 1). Therefore, this analysis includes the 1,881 participants from visit 2 who had no missing data from any of the CVD risk factors or functional measures (N missing = 582 functional, 71 ultrasound, 54 covariates). The remaining participants included 138 in the proband generation (age range 56–106 years) and 1,743 in the offspring generation (age range 42–93 years). Detailed characteristics of the cohort have been described elsewhere^25^. All study forms were approved and methods were performed in accordance with the relevant guidelines and regulations of the Institutional Review Boards at Boston University, Columbia University, and the University of Pittsburgh, and by the Research Ethics Committee at the University of Southern Denmark. Written informed consent to participate in the LLFS and to publish any related research findings was obtained from each LLFS participant.

### Cardiovascular disease risk factors data collection

Carotid measures, including mean intima-media thickness (IMT) and inter-adventitial diameter (IAD), were obtained via B-mode ultrasound of the right and left common carotid arteries, as previously described^18^. Briefly, a GE LOGIQ 3 BT12 Ultrasound System was used to conduct the imaging by centrally certified and trained researchers. A re-scan protocol (N = 10) was conducted per technician to ensure reproducibility and accuracy. Systolic and diastolic blood pressure (SBP and DBP, respectively) were obtained sitting with an automated blood pressure machine and averaged over three measurements (BP-tru BPM 300, VMS MedTech, Coquitlam, Canada). For BMI, height was measured using a Handi-stat set (Perspective Enterprises, Portage, MI) to the nearest 0.1 cm and weight was determined using an electronic scale (SECA 841, Hanover, MD). BMI was calculated as weight(kg)/height(m^2^). Waist circumference (cm) was measured at the umbilicus while the participant stood erect using a soft tape.

### Functional measure data collection

Cognitive function tests included the digit symbol substitution test (DSST), the number span test, the category (animal) fluency test, logical memory tests, and trail making tests. The DSST is used to measure a range of general cognitive operations including executive functions such as planning and strategizing, psychomotor speed, attention, and visuoperceptual functions such as manual dexterity ^26^. The number span test estimates verbal working memory (i.e., short-term memory) which is often used in everyday tasks such as remembering a telephone number or understanding long sentences^27^. The animal fluency test provides a measure of semantic fluency, by asking participants to say aloud all the animals they can within 60 s. Two logical memory tests (IA and IIA) were administered to measure retained memory by asking the participant to immediately recall a story verbatim (immediate recall) and then recall the same story after 20 to 30 min (delayed recall)^28^, the percent which delayed memory score matches immediate score comprises the retained memory variable. The trail making tests (part A and part B) were used to measure psychomotor speed and visual scanning ability (Trails A), and executive functioning, specifically set-switching (Trails B)^29–33^.

Physical function measurements included grip strength, gait speed, and chair stand time. Grip strength was calculated using an isometric dynamometer (Jamar Hydraulic Hand Dynamometer, Lafayette, IN) over an average of two measurements and was rounded to the nearest 2 kg. Gait speed is reported in m/s and is the time required to walk 15 m in a straight-line, on a level indoor surface^34^. Chair stand time (s) is how long it takes an individual to stand up from a straight-back chair without any assistance (i.e., not using their arms).

Additionally, information on each participant’s current smoking status was collected via interviewer-administered questionnaire.

### Statistical analysis

The Sequential Oligogenic Linkage Analysis Routines (SOLAR) program accounts for family structure (i.e., relatedness) using maximum-likelihood based methods to estimate the residual genetic heritability (h^2^_r_) of outcome measures, as well as, the variance attributable to fixed covariate effects^35^. SOLAR first estimates the variance in a trait due to the included covariates (e.g., what is the proportion of the variation in the trait that can be attributed to the covariates?). Then it takes the residual unexplained variance in the trait and estimates the proportion of that attributed to inherited genetic variation (residual genetic heritability; h_2_) based on the input family structure. SOLAR can also estimate the correlation due to shared genetic (i.e., pleiotropy; ρ_G_) or shared environmental (ρ_E_) determinants for pairs of outcomes^35,36^. Phenotypic correlation (ρ) between trait pairs can then be estimated based on the residual heritability and the genetic and environmental correlations. In addition to accounting for family structure, we adjusted all models of carotid measures and blood pressure for age, age^2^, sex, field centers, height, weight, and smoking, as they were significant predictors of the outcomes of interest and not an over-adjustment when considering the likely pleiotropic effects. To avoid multicollinearity, models for obesity (BMI and waist circumference) did not include adjustment for weight (BMI also did not include height adjustment). Where needed, outcome variables were transformed to approximate normality before analysis in SOLAR.

### Ethics approval

All study forms were approved and methods were performed in accordance with the relevant guidelines and regulations of the Institutional Review Boards at Boston University, Columbia University, and the University of Pittsburgh, and by the Research Ethics Committee at the University of Southern Denmark

### Informed consent

Written informed consent to participate in the LLFS and to publish any rela3ted research findings was obtained from each LLFS participant.

## Results

The mean ages in both the proband (N = 138) and offspring (N = 1,743) generations were 89.43 $\pm$ 7.3 years and 67.83 years$\pm 7.6$, respectively, with an overall average age of 69.4 $\pm 9.5$ (Table 1). Within the proband and offspring generations, 41% and 46% of the participants were female, respectively (Table 1). The proband generation has a notably larger IMT (1.01 $\pm$ 0.16 cm), IAD (8.29 $\pm$ 0.76 cm) and SBP (140.91 $\pm$ 20.94 mmHg) than the offspring generation who yielded an average IMT, IAD and SBP of 0.83 cm, 7.69 cm, and 133.39 mmHg, respectively. After adjustment, all studied outcome measures were significantly heritable (range: 0.13 for SBP to 0.62 for IAD; all p $\le$ 0.01; Table 2). Model covariates explained between 4.77% (BMI) and 71% (grip strength) of the variance in outcome measures.

**Table 1:** Characteristics of LLFS Participants.

	Overall (N = 1,881)	Probands (N = 138)	Offspring (N = 1,743)
Age (years)	69.42 (9.46)	89.43 (7.31)	67.83 (7.62)
Female (%)	45%	41%	46%
Current Smoking (%)	4.2%	2.2%	4.4%
Height (cm)	167.17 (9.78)	159.35 (9.10)	167.79 (9.56)
Weight (kg)	76.63 (16.11)	67.72 (12.59)	77.34 (16.15)
Waist (cm)	96.16 (12.86)	95.18 (10.38)	96.24 (13.0)
BMI (kg/m^2^)	27.33 (4.81)	26.57 (3.84)	27.39 (4.88)
Carotid IMT (mm)	0.840 (0.15)	1.01 (0.16)	0.83 (0.14)
IAD (mm)	7.73 (0.86)	8.29 (0.76)	7.69 (0.86)
SBP (mmHg)	133.39 (17.85)	140.91 (20.94)	132.79 (17.45)
DBP (mmHg)	74.64 (10.37)	67.47 (10.00)	75.21 (10.18)

*Characteristics shown as mean (SD) or frequency, as appropriate.

**Table 2:** Residual Genetic Heritability Estimates.

	Residual Heritability Estimate*	Residual Heritability *P*-value	Variance explained by covariates*
**CVD risk factors**			
Carotid mean IMT (mm)	0.478	2.83 × 10^–17^	0.399
Carotid mean IAD (mm)	0.621	3.94 × 10^–22^	0.410
SBP (mmHg)	0.133	0.010	0.111
DBP (mmHg**)**	0.142	0.004	0.252
BMI (kg/m^2^)^#^	0.500	6.99 × 10^–17^	0.477
Waist circumference (cm)^#^	0.494	7.63 × 10^–18^	0.133
**Cognitive function**			
DSST	0.417	5.38 × 10^–15^	0.490
Retained memory	0.285	4.63 × 10^–8^	0.213
Working memory	0.518	2.25 × 10^–17^	0.280
Animal fluency	0.397	4.77 × 10^–13^	0.255
Time to complete trails A	0.220	6.8 × 10^–06^	0.437
Time to complete trails B	0.319	1.0 × 10^–07^	0.464
**Physical function**			
Grip strength (kg)	0.454	5.93 × 10^–18^	0.711
Gait speed (m/s)	0.164	0.002	0.542
Chair stand time (s)	0.264	1.1 × 10^–05^	0.255

*Residual heritability estimates shown range from 0–1 and demonstrate the proportion of the variance in the measure that is attributed to genetic factors after adjustment for covariates. Whereas, the variance explained by covariates is the proportion of the variance in the measure that is explained by the included covariates. Covariates for each model include: age, age^2^, sex, field centers, height, weight, and whether an individual currently smokes.

^#^Weight and height not included as covariates in BMI model; weight not included as covariate in waist circumference model.

Phenotypic correlations between CVD risk factors and functional measures are shown in Table 3. BMI had significant phenotypic correlations with both the cognitive and physical functional measures except for retained memory and time required to complete Trails A. Waist circumference was phenotypically correlated with the same functional measures as BMI (Supplemental Table S1). Systolic and diastolic blood pressures had significant correlation with grip strength (ρ = 0.06 for both). IAD was inversely correlated with DSST and gait speed (ρ = − 0.05 for both) but positively correlated with chair stand time (ρ = 0.05). However, there was no phenotypic correlation between IMT and any functional measure.

**Table 3:** Adjusted phenotypic correlations between CVD risk factors and cognitive and physical function.

	Phenotypic correlation: ρ (*P*-value)				
	Carotid IMT	Carotid IAD	SBP	DBP	BMI*
**Cognitive function**					
DSST	− 0.042 (0.06)	− **0.052 (0.02)**	− 0.030 (0.16)	− 0.033 (0.19)	− **0.102** (< 0.01)
Retained memory (Delayed/Immediate)	− 0.003 (0.91)	− 0.003 (0.89)	− 0.044 (0.05)	− 0.018 (0.43)	− 0.003 (0.89)
Working memory	− 0.013 (0.56)	0.024 (0.30)	− 0.019 (0.38)	− 0.026 (0.24)	− **0.071 (< 0.01)**
Animal fluency	− 0.003 (0.88)	0.005 (0.82)	− 0.023 (0.35)	− 0.017 (0.44)	− **0.067 (< 0.01)**
Time to complete trails A	− 0.009 (0.68)	0.010 (0.66)	0.026 (0.22)	0.034 (0.11)	0.030 (0.17)
Time to complete trails B	0.024 (0.29)	0.012 (0.61)	0.036 (0.10)	0.032 (0.14)	**0.075 (< 0.01)**
**Physical function**					
Grip strength	− 0.035 (0.12)	− 0.034 (0.12)	**0.059 (0.01)**	**0.063 (< 0.01)**	**0.045 (0.04)**
Gait speed	− 0.026 (0.22)	− **0.048 (0.03)**	0.008 (0.70)	− 0.006 (0.79)	− **0.225 (< 0.01)**
Chair stand time	0.013 (0.56)	**0.051 (0.02)**	− 0.025 (0.27)	− 0.034 (0.13)	**0.173 (< 0.01)**

Values shown are correlation estimates (*P*-values) between CVD risk factor and cognitive or physical function measure as estimated by SOLAR. **BOLD** indicates a statistically significant correlation (*P* < 0.05).

Covariate adjustments : age, age^2^, sex, field centers, height, weight, and whether an individual currently smokes.

*BMI models do not include adjustment for height or weight.

Genetic correlations between CVD risk factors and functional measures are shown in Table 4). There were significant (*P* < 0.05) genetic correlations between DSST and SBP (ρ_G_ = − 0.37), DBP (ρ_G_ = − 0.61), and BMI (ρ_G_ = − 0.21). Retained memory was genetically correlated with SBP (ρ_G_ = − 0.45) and BMI (ρ_G_ = − 0.34); whereas, working memory was genetically correlated with IMT (ρ_G_ = − 0.26). Animal fluency was genetically correlated with systolic (ρ_G_ = − 0.40) and diastolic (ρ_G_ = − 0.45) blood pressures. The time required to complete Trails A was genetically correlated to diastolic (ρ_G_ = 0.49) but not, systolic blood pressure. Both gait speed and chair stand time were genetically correlated with BMI (ρ_G_ = − 0.40 and ρ_G_ = 0.29, respectively). Genetic correlations for waist circumference (Supplemental Table S1) were largely similar to those for BMI. In all cases, significant genetic correlations were significantly different from both 0 and 1, suggesting a portion but not all of their genetic covariance was shared between the trait pairs.

**Table 4:** Adjusted genetic correlations (ρ_*G*_) between CVD risk factors and cognitive and physical function.

	Carotid IMT		Carotid IAD		SBP		DBP		BMI*	
	ρ_G_	P_0_ | P_1_	ρ_G_	P_0_ | P_1_	ρ_G_	P_0_ | P_1_	ρ_G_	P_0_ | P_1_	**ρ**_**G**_	**P**_**0**_** | P**_**1**_
**Cognitive function**										
DSST	− 0.015	0.88 | 5.6 × 10^–15^	− 0.026	0.79 | 5.9 × 10^–15^	− **0.374**	**0.04 | 0.02**	− **0.610**	**3.0 × 10**^**–4**^** | 0.049**	− **0.206**	**0.049 | 2.1 × 10**^**–12**^
Retained memory (Delayed/Immediate)	0.110	0.40 | 2.0 × 10^–7^	0.065	0.12 | 3.0 × 10^–7^	− **0.454**	**0.04 | 0.04**	− 0.355	0.09 | 0.01	− **0.340**	**0.01 | 1.7 × 10**^**–5**^
Working memory	− **0.264**	**0.01 | 2.2 × 10**^**–10**^	0.064	0.49 | 2.3 × 10^–17^	− 0.236	0.19 | 0.02	− 0.121	0.48 | 0.005	− 0.165	0.10 | 3.3 × 10^–15^
Animal fluency	0.054	0.62 | 1.8 × 10^–10^	0.089	0.39 | 9.1 × 10^–12^	− **0.396**	**0.04 | 0.03**	− **0.445**	**0.02 | 0.02**	− 0.142	0.20 | 7.4 × 10^–12^
Time to complete trails A	− 0.071	0.60 | 9.2 × 10^–6^	− 0.034	0.79 | 7.0 × 10^–6^	0.168	0.48 | 0.01	**0.485**	**0.03 | 0.04**	0.140	0.30 | 9.4 × 10^–6^
Time to complete trails B	0.083	0.51 | 2.0 × 10^–7^	− 0.071	0.55 | 2.0 × 10^–7^	0.180	0.43 | 0.01	0.264	0.21 | 0.006	0.221	0.08 | 6.0 × 10^–7^
**Physical function**										
Grip strength	− 0.089	0.38 | 1.2 × 10^–14^	− 0.008	0.93 | 2.6 × 10^–16^	− 0.238	0.21 | 0.03	− 0.277	0.11 | 0.01	0.002	0.99 | 1.2 × 10^–17^
Gait speed	0.010	0.95 | 0.002	0.135	0.39 | 0.003	0.027	0.93 | 0.02	− 0.217	0.42 | 0.008	− **0.404**	**0.01 | 0.003**
Chair stand time	− 0.064	0.64 | 1.0 × 10^–5^	− 0.010	0.94 | 1.3 × 10^–5^	0.257	0.32 | 0.02	0.198	0.39 | 0.007	**0.294**	**0.03 | 6.2 × 10**^**–6**^

Values shown are estimates of the correlation between CVD risk factor and cognitive or physical function measure that is due to genetic factors (e.g., genetic correlation (**ρ**_**G**_). *P*-values are shown both for the test for a significant difference from zero (0) (i.e., some shared genetic variance; P_0_) and the test of a significant difference from unity (1) (i.e., completely shared genetic variance; P_1_). To facilitate quick interpretation, all instances of significant genetic correlation are shown in **BOLD.**

Covariate adjustments: age, age^2^, sex, field centers, height, weight, and whether an individual currently smokes.

*BMI models do not include adjustment for height or weight.

Environmental correlations, i.e. the correlation due to the included covariates, between CVD risk factors and functional measures are shown in Supplemental Table S2. These results show that environmental factors in these models (age, sex, field center, smoking status, and body size) showed significant covariance between CVD risk factors and measures of both cognitive and physical functioning.

## Discussion

**Figure 2:**
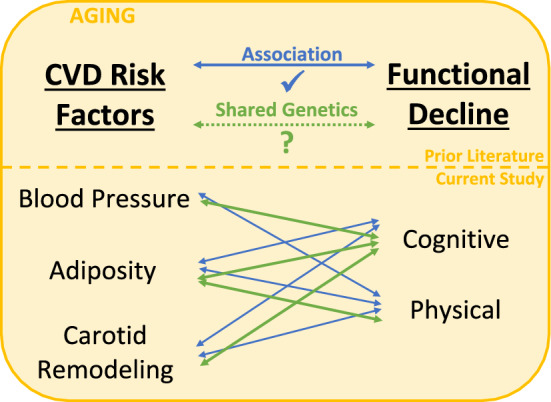
Summary Diagram of the Current Study’s Purpose and Findings. Infographic of the study purpose (dashed green line, top portion of figure) and findings (bottom portion of figure), including highlighting trait pairs with significant phenotypic (blue lines) or genetic (green lines) correlations.

The genetic correlation results from this study in long-lived families provide novel evidence of shared genetic variance between cognitive function and CVD risk factors , as well as, between measures of physical function and obesity (Fig. 2). The study is also consistent with existing literature that demonstrated a phenotypic correlation between lower physical function and adverse CVD risk factors. The significant pleiotropic results suggest that CVD is not only a risk factor for cognitive and physical decline, but may also be partially driven by the same genes and have a shared pathology.

The relationship between BMI and physical function is known to differ by age, such that a greater BMI in early to middle adulthood is associated with an increased risk of chronic disease and mortality^37^, but in geriatric populations, greater BMI is associated with reduced frailty and a lower risk of morbidity and mortality^38^. Not only does obesity in early to middle adulthood appear to increase mortality risk, but it also prompts declines in physical function and may even lead to overt frailty and it subsequent outcomes^39,40^. In our study, both gait speed and chair stand time were genetically and phenotypically correlated with obesity (e.g., BMI, waist circumference), but not cardiovascular measures (e.g., IAD, IMT, SBP, DBP). This relationship is consistent and expands upon previous phenotypic correlation studies of obesity and frailty.

In addition to its shared genetic variance with physical function, we also found that BMI shares genetic variance with some measures of cognitive function. All of the cognitive measures that were genetically correlated with BMI approximate frontal lobe function, an area of the brain that is responsible for actions such as executive functioning and is susceptible to the detrimental effects of aging^41,42^. These novel findings should motivate researchers to investigate the shared pathophysiology between CVD risk factors, obesity in particular, and aging-related functional decline.

Unlike BMI, measures of subclinical carotid vessel disease were not genetically correlated with measures of physical function, although there were some significant phenotypic correlations. These phenotypic results are consistent with previous findings in healthy aging cohorts. In the Health ABC study, researchers found that both peripheral artery disease and subclinical atherosclerosis were cross-sectionally associated with walking speed after adjustment for potential confounders^43^. They also demonstrated that while frailty may precede 10-year heart failure incidence^44^, arterial fibrillation may actually precede and contribute to longitudinal physical decline^45^. This inconsistency in the temporal relationship between carotid vessel disease and physical function highlight a need for further research and, since our study did not find evidence of a shared genetic pathway between carotid vessel disease and physical function, the potential shared environmental determinants of these disorders should be of particular focus.

In contrast, measures of subclinical carotid vessel disease showed significant genetic correlation with cognitive measures, specifically those that approximate working memory, executive functioning and semantic fluency performance. Our results align with previous studies which demonstrate a longitudinal association between CVD and cognitive decline^10–16^. However, unlike physical function, which showed both strong phenotypic and genetic correlations with CVD risk factors , cognitive function had significant genetic, but not phenotypic, correlation with CVD risk factors. One potential explanation could be that phenotypic correlation is an aggregate of both the genetic and environmental correlation values. For example, take IMT and working memory: their genetic correlation is negative, but the environmental correlation is positive (Supplemental Table S2), thus resulting in an insignificant phenotypic correlation. This incongruence between the direction of genetic and environment effects may be partially driven by the wide age-range of our participants. When we exclude age as a covariate from the model, the environmental correlation effect becomes negative, thus mirroring the direction of the genetic correlation. While there is no procedure to test for interactions or modification in analyses of genetic correlation using a family study design, future research should be conducted in narrow age ranges in order to identify age-specific directionality of the genetic and phenotypic relationship between cognitive function and carotid vessel disease. It is possible that there is a non-linear relationship of carotid vessel disease and cognitive function, similar to that of BMI and physical function declines discussed above.

One important limitation is that due to the cross-sectional nature of this study, we cannot assess temporality, meaning we can’t know for sure whether declines in function preceded, succeeded or occurred concurrently with the incidence of CVD. Also, since the main objective of the LLFS is to discover factors associated with exceptional longevity, the older-lived subjects in this study may have been healthier across their lifespan compared to the general population^25,46,47^. Thus, it is possible that they harbor unique genetic interactions or pleiotropic effects that may not apply to the general population. However, the estimation of genetic correlation requires a family-study design. As such, given the extensive outcome measurements and unique design, the LLFS is ideally suited to perform these analyses. Additionally, because this is a family-based study, family members likely experience shared lifestyles or environments for at least a portion of their lives, and therefore, we may be at risk of overestimating the genetic correlation and heritability of certain traits^48,49^. Since our primary goal was to estimate the genetic correlation between CVD risk factors and age-related functional measures, our focus was on including adjustment for biologic covariates outside of the biologic pathways of interest. However, it is possible that there may be some environmental covariates such as education and depression^5,10,12,15^ that we did not include in this study, which could be important particularly for phenotypic correlation results. Also, it is known that there is a tight relationship between obesity and type 2 diabetes, such that obesity is considered a precursor for type 2 diabetes^50^. Therefore, we did not consider type 2 diabetes or other CVDs (e.g., dyslipidemia, heart failure, stroke) as covariates because they are in the causal pathway between CVD risk factors and functional decline, it would be an over-adjustment in estimating the proportion of their shared genetic variance. However, we believe that these other comorbidities and CVDs are important to consider when evaluating the relationship between CVD risk factors and functional decline.

The overall findings of our study suggest that CVD risk factors may share genetic determinants with both cognitive and physical function, thus suggesting there may be common physiological mechanisms linking these age-related disorders. These results help to improve our understanding of the underlying biologic links between aging-related conditions and may prompt innovations in geriatric care, particularly in those afflicted with cardiovascular disease. The next step for this research should attempt to replicate and extend these findings in other studies that are reflective of the general adult population, as well as, attempting to identify genes and/or pathways responsible for the pleiotropy between these measures in the LLFS.

## Supplementary Information

Supplementary Information.

## Data Availability

All data used in this analysis is available at dbGAP (accession phs000397.v2.p2).

## Code availability

All analyses were conducted using standard code using the Sequential Oligogenic Linkage Analysis Routines (SOLAR) program.
