# Supplementary material for: Pleiotropic effects between cardiovascular disease risk factors and measures of cognitive and physical function in long-lived adults
Source: Sci Rep. 2021 Sep 9;11:17980. doi: 10.1038/s41598-021-97298-0 (PMC8429644; doi:10.1038/s41598-021-97298-0)
Supplement: Supplementary file 1 — Supplementary Information. [file 41598_2021_97298_MOESM1_ESM.docx]

**Supplemental Material**

**Table S1. Phenotypic and Genetic Correlations between Waist and Cognitive and Physical Function**

| **Functional Variables** | **Phenotypic Correlation ρ** | **Genetic Correlation** | |
| --- | --- | --- | --- |
|  |  | **ρ_G_** | **P_0.0_ \| P_1.0_** |
| **Cognitive Function** | | | |
| DSST | **-0.112** | -0.19 | 0.07 \| 5.9x10^-13^ |
| Retained Memory | 0.002 | -0.177 | 0.175 \| 5.0x10^-7^ |
| Working Memory | **-0.079** | -0.17 | 0.09 \| 6.0x10^-16^ |
| Animal Fluency | **-0.099** | -0.165 | 0.14 \| 4.8x10^-12^ |
| Time to Complete Trails A | 0.009 | 0.548 | 0.06 \| 0.28 |
| Time to Complete Trails B | **0.063** | **0.256** | **0.11 \| 0.002** |
| **Physical Function** | | | |
| Grip Strength | **0.045** | -0.023 | 0.82 \| 5.7x10^-14^ |
| Gait Speed | **-0.229** | **-0.343** | **0.034 \| 0.002** |
| Chair Stand Time | **0.229** | **0.525** | **3.6x10^-4^ \| 0.002** |

Covariate Adjustments for waist size: age, age^2^, sex, field centers, height, and whether an individual currently smokes

Values in bold indicate a statistically significant correlation value (p<0.05)

**Table S2. Environmental Correlations between Cardiometabolic Traits and Cognitive and Physical Function**

|  | **Environmental Correlation: ρ_E_ (SE)** | | | | | |
| --- | --- | --- | --- | --- | --- | --- |
|  | **IMT** | **IAD** | **SBP** | **DBP** | **BMI** | **Waist** |
| **Cognitive Function** | | | | | | |
| DSST | -0.064 (0.076) | -0.081 (0.092) | 0.087 (0.063) | **0.173 (0.062)** | -0.015  (0.080) | -0.049 (0.076) |
| Retained Memory | -0.07  (0.075) | -0.014 (0.081) | 0.066 (0.073) | -0.071 (0.058) | **-0.200 (0.078)** | 0.113 (0.074) |
| Working Memory | **0.231 (0.089)** | -0.030 (0.108) | 0.063 (0.060) | 0.011 (0.070) | 0.031 (0.094) | 0.011 (0.090) |
| Animal Fluency | -0.048 (0.079) | -0.158 (0.090) | 0.098 (0.064) | **0.125 (0.063)** | -0.006 (0.083) | -0.048 (0.078) |
| Time to Complete Trails A | 0.022 (0.065) | -0.080 (0.095) | -0.001 (0.053) | -0.064 (0.052) | -0.029  (0.070) | -0.082 (0.050) |
| Time to Complete Trails B | -0.014 (0.077) | 0.085 (0.094) | -0.003 (0.062) | -0.032 (0.060) | -0.028 (0.082) | -0.003 (0.060) |
| **Physical Function** | | | | | | |
| Grip Strength | -0.011 (0.077) | -0.065 (0.093) | **0.166 (0.062)** | **0.189 (0.060)** | 0.087 (0.085) | 0.106 (0.082) |
| Gait Speed | -0.044 (0.067) | **-0.160 (0.081)** | 0.006 (0.054) | 0.034 (0.053) | **-0.165 (0.069)** | **-0.200 (0.066)** |
| Chair Stand Time | 0.059 (0.076) | 0.105 (0.091) | -0.091 (0.620) | -0.092 (0.059) | 0.102 (0.081) | 0.094 (0.071) |

Values in **BOLD** indicate a statistically significant correlation estimate (p$\leq$0.048)

Covariate adjustments for carotid and BP: age, age^2^, sex, field centers, height, weight, and whether an individual currently smokes;

Covariate Adjustments for BMI: age, age^2^, sex, field centers, and whether an individual currently smokes

Covariate Adjustments for Waist: age, age^2^, sex, field centers, height, and whether an individual currently smokes
